# Supplementary figures and images for: Different revegetation types alter soil physical-chemical characteristics and fungal community in the Baishilazi Nature Reserve
Source: PeerJ. 2019 Jan 11;6:e6251. doi: 10.7717/peerj.6251 (PMC6330947; doi:10.7717/peerj.6251)

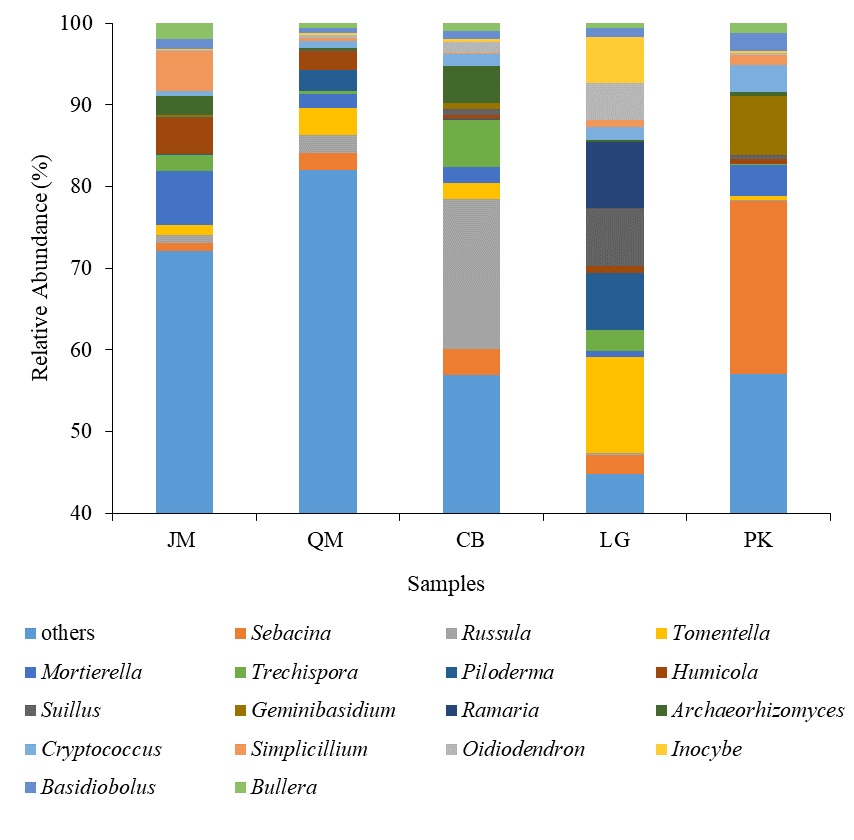

Supplement: Supplemental Information 6 — JM: Juglans mandshurica; QM: Quercus mongolica; CB: Conifer-broadleaf forest; LG: Larix gmelinii; PK: Pinus koraiensis. [file peerj-07-6251-s006.png]
